# Robust and Fragile Medical Image Watermarking: A Joint Venture of Coding and Chaos Theories

**DOI:** 10.1155/2018/8137436

**Published:** 2018-07-02

**Authors:** Atta Ur Rahman, Kiran Sultan, Dhiaa Musleh, Nahier Aldhafferi, Abdullah Alqahtani, Maqsood Mahmud

**Affiliations:** ^1^Department of Computer Science, College of Computer Science and Information Technology, Imam Abdulrahman Bin Faisal University (IAU), P.O. Box. 1982, Dammam, Saudi Arabia; ^2^Department of CIT, JCC, King Abdulaziz University, Jeddah, Saudi Arabia; ^3^Department of Computer Information System, College of Computer Science and Information Technology, Imam Abdulrahman Bin Faisal University (IAU), P.O. Box. 1982, Dammam, Saudi Arabia; ^4^Department of MIS, College of Business Administration, Imam Abdulrahman Bin Faisal University (IAU), P.O. Box 1982, Dammam, Saudi Arabia

## Abstract

A secure spatial domain, hybrid watermarking technique for obtaining watermark (authentication information) robustness and fragility of the host medical image (content integrity) using product codes, chaos theory, and residue number system (RNS) is proposed. The proposed scheme is highly fragile and unrecoverable in terms of the host image, but it is significantly robust and recoverable in terms of the watermark. Altering the medical image may result in misdiagnosis, hence the watermark that may contain patient information and organization logo must be protected against certain attacks. The host medical image is separated into two parts, namely, the region of interest (ROI) and region of noninterest (RONI) using a rectangular region. The RONI part is used to embed the watermark information. Moreover, two watermarks are used: one to achieve authenticity of image and the other to achieve the robustness against both incidental and malicious attacks. Effectiveness in terms of security, robustness, and fragility of the proposed scheme is demonstrated by the simulations and comparison with the other state-of-the-art techniques.

## 1. Introduction

Digital watermarking is one of the most promising techniques for authentication, copyright protection, and ownership identification. In digital watermarking, the watermark information could be fragile, semifragile, robust, or hybrid. Fragile watermarks are very sensitive and are used for tamper detection, while the robust watermarks are used to withstand common image processing operations. The watermark having robustness against friendly attacks while fragile against malicious attacks is called semifragile, and the one having mixed properties is called a hybrid watermark [1]. Medical image watermarking is becoming more promising in terms of security, privacy, and authenticity; hence, several schemes have been investigated in this regard. Major purposes of the medical image watermarking are authenticity and integrity control. Here, the authenticity is referred to as the measure through which it is ensured that the image source is valid and the image belongs to the right patient. The integrity control is a capability to validate that image has not been tampered by any means. Since, in some cases, little modifications in the medical image may cause a wrong diagnosis [2, 3], the authors in [4] focused on integrity control and authentication of patient electronic record (PER). While in [5], the authors focused on three areas in medical image watermarking that are authentication, data hiding, and their combination. In this paper, a DICOM image was selected for experiments, after being divided into ROI and RONI parts. The major drawback of the technique was fragility of the watermark. Because the PER is an important information, it must not be preserved through the fragile methods that make it vulnerable against many attacks. In [6], the authors proposed an integrity verification and authentication of a medical image (ultrasound) by considering it from DICOM image bank. The ROI was divided by a rectangular region from the RONI area. Then, the hash value of the whole image was calculated by means of hash function SHA-256. For the sake of enhancing the security, two secret keys were used: one as the hash key and the other as the embedded watermark key. Eventually, the watermark was embedded in the LBS of the RONI area. The received image was assumed authenticated if it possessed a high degree of correlation. Akin to [6], in [7], the authors also selected the RONI as watermarking area for authentication purpose. However, the reversibility was not ensured, if the RONI was altered. In [8–10], the authors proposed a reversible watermarking scheme for medical images. The scheme is secure but exhibits a very low level of imperceptibility. The authors proposed a blind hybrid watermarking technique for medical images in [11]. The first watermark was made robust that consisted of doctor ID, patient information, and LSBs map of ROI which were embedded into the RONI after encryption. The second watermark was used for the sake of fragility (integrity control). It consisted of a binary pattern and was embedded inside the ROI. The results show that the proposed scheme does not provide a good measure of hybridization. In [12], the authors presented a fragile image watermarking technique using SVD characteristics for authentication. Akin to this, in [13], the authors presented a perceptual hash algorithm for multispectral image authentication. In [14], the authors presented a semifragile, blind image authentication using discrete curvelet transform. The scheme was promising in terms of robustness and high normalized correlation. In [15, 16], the authors proposed techniques to optimize the three conflicting parameters of image watermarking. However, the techniques are not suitable for medical images in general. In [17], the authors provide a secure transform domain watermarking scheme using error correcting codes. The scheme exhibits good protection against various attacks including checkmark and channel noise. A similar approach has been carried out in [18] where the authors utilized product codes and cubic product codes for watermark protection in spatial domain for medical images. However, there is no detail provided regarding imperceptibility and capacity. In [19], the authors proposed a spread spectrum-based medical image watermarking technique. The scheme provided high level of fragility, but the capacity and imperceptibility of the scheme were not up to the mark. In [20], the authors provided a medical image authentication technique using RNS and chaos theory. However, the scheme exhibits a very poor imperceptibility. Yu et al. [21] presented a comprehensive review of recoverable and nonrecoverable semifragile watermarking techniques on medical images in spatial and transform domain.

It is concluded that there does not exist a perfect semifragile watermarking technique that can distinguish between the friendly (image processing) and malicious attacks. Moreover, it is hard to balance between fragility and robustness [21]. Similarly, there does not exist a technique that results in a robust watermark (authentication) and a perfectly fragile host medical image that does not tolerate on a single pixel tamper (100% content integrity). Because that pixel/s may be important for some diagnostics such as tumor detection. The situation becomes even critical if the watermark information is significant in size compared to the host image and a considerable level of imperceptibility is to be achieved. To overcome these issues, in this paper, a spatial domain, secure, hybrid watermarking technique for making the watermark (patient information/organization logo) robust and the host medical image highly fragile (content integrity) using product codes, chaos theory, and RNS is proposed. The technique is unrecoverable in terms of the host image; however, the watermark is recoverable because of error correcting codes (ECC). The image is divided into the region of interest (ROI) and the region of noninterest (RONI) using the smallest rectangular area. Then, based on a chaotic key, some precalculated pixels in RONI are made zero. Then, this RONI is rearranged with residued ROI, and a hash is generated for the whole image which acts as a basis of a fragile watermark. This will give us 128 bits. On the contrary, the watermark is converted to redundant residues and then encoded using error correcting codes to achieve robustness and is concatenated with fragile watermark. Again, based on the same chaotic key, concatenated watermark is embedded in RONI pixels by replacing its 4 LSBs. On the receiver side, based on the chaotic key, both watermarks are extracted. Hence, the chaotic key provides the means of security.

The rest of the paper is divided as follows: Section 2 introduces the techniques used.  Section 3 contains the proposed technique.  Section 4 contains simulation results, while Section 5 concludes the paper.

## 2. Techniques Applied

### 2.1. Chaotic Systems

Chaotic systems exhibit many characteristics that differentiate them from the other systems. One characteristic is their sensitivity to the initial conditions which if slightly modified, an entirely different pattern is obtained. Hence, a chaotic system can be used as a pseudonoise (PN) generator. It means many nonperiodic and noise-like sequences can be generated. One of the simplest maps is logistic map which has a recursive relation that describes population growth over the time which is given as

(1) $$\begin{array}{c} X_{n}+1=rX_{n}\left(1-X_{n}\right), \end{array}$$

where *r* is a bifurcation parameter and 3.57 < *r* ≤ 4 for the system to be chaotic and *x*_0_ belongs to (0, 1). Once the sequence of real numbers is generated, a binary sequence is produced with approximately equal number of 1's and 0's.

### 2.2. Hash Function

**Figure 1:**
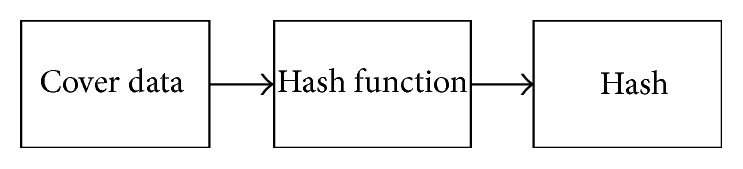
Generic diagram for hashing.

Hash is a one-way function that can produce a signature called a message digest/hash value after applying on the data. Reverse never happens that given the hash value, it computes the data as depicted in Figure 1. This, one-way property of hash algorithm can be used for authenticity of data. Secure hash algorithm (SHA-256) is also a kind of hashing function which gives 256-bit message digest.

### 2.3. Product Codes

**Figure 2:**
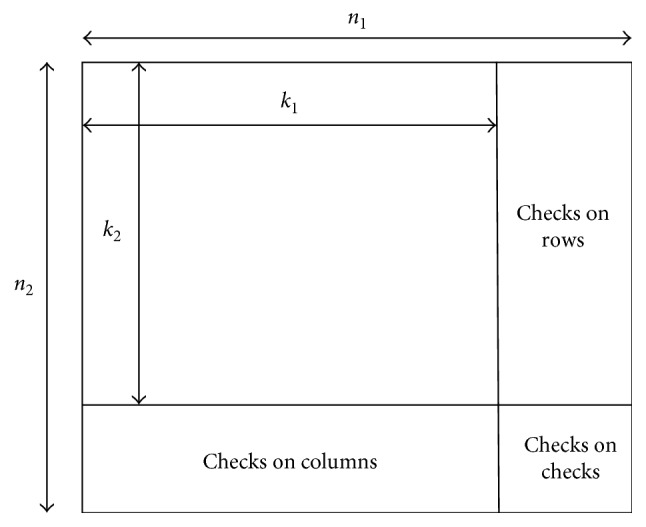
Structure of product codes.

Product codes are two-dimensional codes where each dimension contains block codes. Due to their unique arrangement, product codes have capability of correcting random errors as well as burst errors since any upcoming error in row-wise can be easily corrected through column-wise and vice versa. Since burst error occurring in rows will become single error for column code and vice versa so, by using these codes, the error correcting capability can be enhanced, as shown in Figure 2. More specifically, the product code can be easily understood in the following manner. In this work, a low complexity decoder of the product codes is utilized [22, 23]. Suppose we have two block codes A_1_ with attributes (*n*_1_, *k*_1_, and *d*_1_) and A_2_ with attributes (*n*_2_, *k*_2_, and *d*_2_), where *n*_*i*_, *k*_*i*_, and *d*_*i*_ are code word length, message size, and minimum distance of constituent codes, respectively:

1. Place an array of *k*_1_ × *k*_2_ information bits with *k*_2_ columns and *k*_1_ rows as shown in Figure 2
2. Encode *k*_2_ rows using code *A*_1_, which will result in an array of *k*_2_ × *n*_1_
3. Now encode *n*_1_ columns using code A_2_, which will result in *n*_1_ × *n*_2_ product code.

### 2.4. Residue Number System (RNS) and Chinese Remainder Theorem (CRT)

A riddle posted in a book by a Chinese scholar called Sun Tzu in the first century was the first documented manifestation of residue number system (RNS) representation as discussed by Grosswald (1996) and Jenkins (1993) [24]. In RNS, any integer *X* can be represented with a small set of integers called residues (*x*_1_, *x*_2_,…, *x*_*i*_), where *x*_*i*_ represents the *i*th residue. The relation can be given as

(2) $$\begin{array}{c} x_{i}=X\;\mathrm{mod}{\;m}_{i}, \end{array}$$

where *m*_*i*_ is the *i*th modulus.

The RNS provides unique representation for all integers in the range between 0 and M. If the integer *X* is greater than *M* − 1, the RNS representation repeats itself. Therefore, more than one integer might have the same residue representation. The dynamic range of RNS is given as follows: *M*=∏_*i*=1_^*k*^*m*_*i*_. It is important to emphasize that the moduli must be relatively prime to be able to exploit the full dynamic range *M*. Given a set of pair-wise relatively prime moduli and a residue representation (*x*_1_, *x*_2_,…, *x*_*k*_) in that system from which residues have been made. Here, the number *X* can be calculated using CRT as follows:

(3) $$\begin{array}{c} X=\left[\sum_{i=1}^{k}M_{i}\left|x_{i}\;L_{i}\right|m_{i}\right]\mathrm{mod}\;M, \end{array}$$

where *M*=∏_*i*=1_^*k*^*m*_*i*_ and |*x*_*i*_*L*_*i*_|*m*_*i*_=1, in which *L*_*i*_ is the multiplicative inverse of *M*_*i*_ with respect to *m*_*i*_.

## 3. Proposed Watermarking Scheme

This section presents the proposed digital watermarking scheme for medical images.

### 3.1. Watermarking Embedding

**Figure 3:**
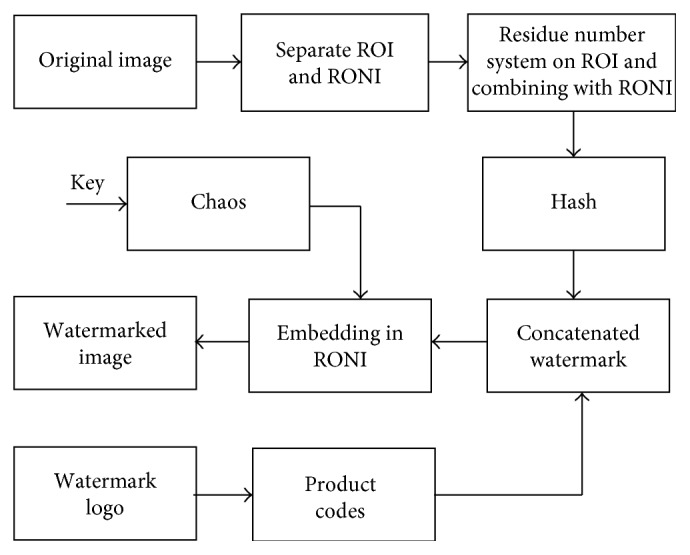
Watermark embedding.

**Figure 4:**
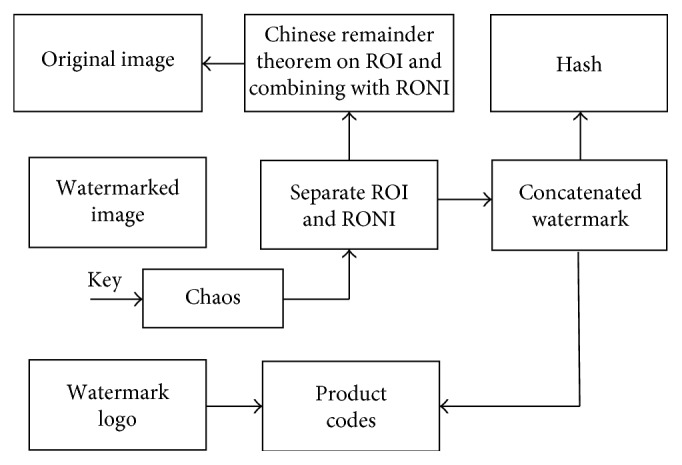
Watermark extraction.

Watermarking embedding and watermark and original image extraction schemes are given in Figures 3 and 4, respectively. Detailed steps of watermark embedding are as follows:

1. Extract the possible ROI from the original image by bounding the smallest rectangle around the desired area, since only the ROI part of the image is to be converted to residues as explained in step 2.
2. The value 255 is factorized to 17 and 15 (relatively prime factors) corresponding to the moduli pair (*m*_1_, *m*_2_) of the RNS to be used for this image. Since the dynamic range of RNS is 0 to 254, every pixel with intensity value 255 is treated separately as explained below. Preprocessing of the pixel being converted to residue is a key to get pixels back. For every pixel of ROI, we get the residue pair (*x*_1_, *x*_2_), where *x*_*i*_ = *X* mod *m*_*i*_ such that *x*_1_ ≤ 16 and *x*_2_ ≤ 14. Except for the case when *x*_1_ = 16, where 5 bits are needed to represent 16, all *x*_*i*_ occupied 4 bits. This makes the pair (*x*_1_, *x*_2_) to be represented over 8 bits. Now to resolve the issue of having those pairs where the first residue *x*_1_ is 16, here, we applied a trick. The pairs having first residue as 16 are mapped to corresponding unique pairs which do not otherwise occur in this RNS scheme. The mapping scheme is given in Table 1. All the pairs which were to be represented by 9 bits each are mapped to the unique pairs which can be represented by 8 bits each. Since 15 cannot occur in the normal pairs as a second residue, it acts as an indicator that these pairs are exceptional pairs. Forward process and inverse process for these exceptional pairs are as follows:
  - Forward process at transmitter end: (16, 12) → (12, 16) → (12, 15)
  - Inverse process at receiver end: (12, 15) → (15, 12) → (16, 12)

(3) Generate a chaotic sequence using (1) and multiply by 6 and take its ceil(.) so that the real chaotic sequence maps into integers as
  (4) $$\begin{array}{c} S=\left\{X_{1},X_{2},X_{3},\ldots \right\}. \end{array}$$

Similarly, pixel 255 has residue (0, 0). We need to differentiate it from pixel 0 which also has residue (0, 0). We send the pixel valued 255 as a pair (15, 15), which can again be represented by 8 bits. This unique pair will not occur in this residual scheme with moduli 17 and 1.

Now change the above sequence into sum sequence as

(5) $$\begin{array}{c} S=\left\{X_{1},X_{1}+X_{2},X_{1}+X_{2}+X_{3},\ldots \right\}\\ =\left\{Y_{1},Y_{2},Y_{3},\ldots ,Y_{n}\right\}, \end{array}$$

where *Y*_*i*_=∑_*n*=1_^*i*^*X*_*n*_.

After changing into sum sequence, divide each sequence by 2 so that its range corresponds within RONI and then take its ceiling as

(6) $$\begin{array}{c} Z=\text{ceil}\left\{\frac{Y_{1}}{2},\frac{Y_{2}}{2},\frac{Y_{3}}{2},\ldots ,\frac{Y_{14207}}{2}\right\}. \end{array}$$

This sequence which is an outcome of chaotic key will give locations for embedding the concatenated watermark in RONI:

(4) Arrange all the pixels of RONI in the vector form using the chaotic sequence obtained in previous step and make the first 4 LSBs of those corresponding to pixel of RONI to zero.
(5) Rearrange the RONI pixels obtained in step 4 with residued ROI obtained in step 2.
(6) Compute the hash of the image obtained in step 5. This will give 128 bits one-way hash value which will be used as an authentication watermark for the image.
(7) Read the second watermark logo which should be made robust and convert it into binary *k*_1_ × *k*_2_ matrix.
(8) Encode the binary watermark logo using product codes which gives us a product encoded watermark.
(9) Arrange the encoded watermark in a vector and concatenate it with the hash computed in previous step and call it a concatenated watermark *W*_c_ as

(7) $$\begin{array}{c} W_{c}=\left[H,W_{e}\right]. \end{array}$$

(10) Again, arrange the pixels of RONI in the vector form as in step 4 and embed the watermark *W*_c_ based on the chaotic key computed in step 3 by replacing the four LSBs of RONI with every 4 bits of the watermark *W*_c_.
(11) Rearrange the RONI pixels in their original position to obtain the watermarked image. Now the concatenated watermark *W*_c_ exists in RONI while the ROI is residued.

### 3.2. Watermark and Original Image Extraction Scheme

Watermark and original image extraction steps are as follows:

(1) Separate the RONI from the ROI in the watermarked image.
(2) Arrange all the pixels of RONI in some arbitrary vector. Now by knowing the chaotic key, watermarked pixels are indicated as
  (8) $$\begin{array}{c} S_{1}=\left\{Y_{1},Y_{2},Y_{3},\ldots ,Y_{n}\right\}. \end{array}$$

This sequence is an outcome of chaotic key which will give locations for extracting the concatenated watermark *W*_c_ from RONI.

(3) Using the chaotic sequence obtained in step 2, extract the concatenated watermark *W*_c_ from the first 4 LSBs of RONI pixels in some vector and place 4 bit values as zero at that location as *W*_c_=[*H*, *W*_e_].
(4) Separate the encoded watermark *W*_e_ from the concatenated watermark *W*_c_ and decode it to get the actual information.
(5) Rearrange these RONI pixels with the residued ROI to form the image.
(6) Rearrange these RONI pixels with the residue converted ROI to form an image.
(7) Now compute the hash of this whole image and store it as hash_2. Also separate the hash from the concatenated watermark *W*_c_ and store it as hash_1.
(8) Compare hash_1 with hash_2. If the hash is same, then the image is authentic, and hence it goes to next steps, otherwise the image is tampered.
(9) In scanning residued ROI, the residue pairs having second residue as 15 go through conversion process as given in the step 2 of embedding. In this way, all of the residue pairs are converted into 9 bits again. Then again apply CRT in (3) to get back original pixels of ROI from residues.
(10) Combine these ROI pixels with RONI pixels to get the original image.

## 4. Simulation Results

**Figure 5:**
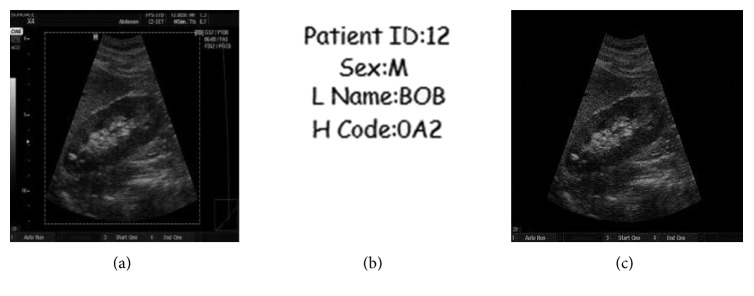
(a) Original ultrasound image. (b) Watermark logo. (c) Watermarked image. Hash of the image: a16c4d371f512de40f836428ea2541fe.

To see the effectiveness of the proposed system, the experiment was conducted in MATLAB. The test image is ultrasound image of size 194 × 259 greyscale pixels, and the watermark logo of size 30 × 30 was considered. BCH codes (127, 99, and 4) were used as row-wise as well as column-wise in the formation of product codes, respectively, for the robustness of the watermark. The proposed watermarking scheme effectively embeds both the watermarks into original image and then extracts both the watermarks from the watermarked image. The logistic map 2 given in (1) with initial conditions *x*(0) = 0.25 and *r* = 3.58 at embedding side and SHA-128 hash algorithm are used to calculate the hash of image.  Figure 5(a) shows the original ultrasound image in which ROI is selected as the smallest rectangular region that bounds the ROI region, and Figure 5(b) shows the watermark logo and Figure 5(c) shows the watermarked image. Hash of the image is given with the figure captions.

### 4.1. Security Analysis

**Figure 6:**
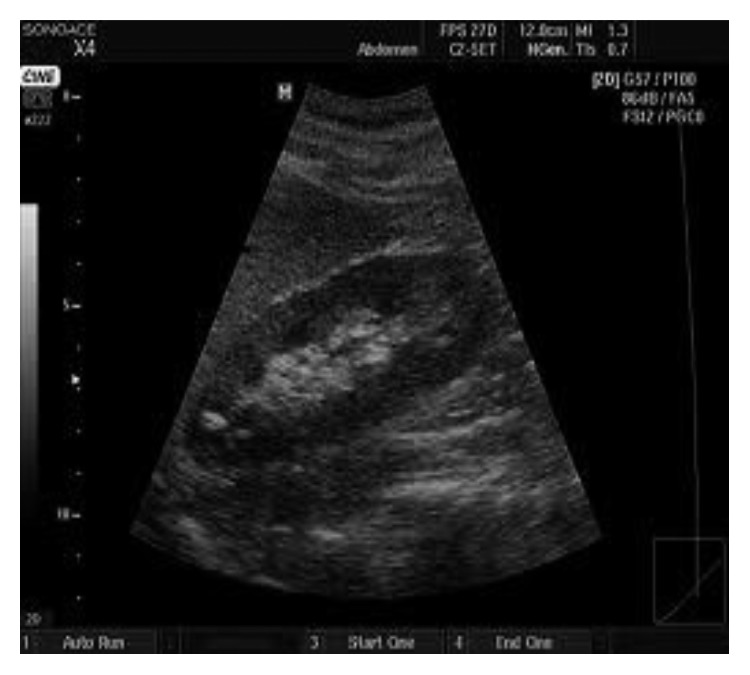
Recovered image using exact initial conditions *x*(0) = 0.25 and *r* = 3.58 at receiver side.

In this experiment, security analysis of the proposed scheme is shown firstly by using same initial conditions of the chaotic system at extracting stage and then by using slightly modifying initial conditions at extracting stage. Exact hash and original image is recovered when no attack is applied to the watermarked image.  Figure 6 shows the recovered image and hash with initial conditions *x*(0) = 0.25 and *r* = 3.58 which are exactly the same as the image shown in Figure 5 and the hash given with Figure 5.  Table 2 shows the recovered hash by using same initial conditions and with slightly modified initial conditions *x*(0) = 0.250001 and *r* = 3.58. In the latter case, exact hash is not recovered, which means the image is tampered. This shows the effectiveness of the proposed scheme in terms of fragility.

### 4.2. Robustness Analysis

This section provides analysis of the proposed scheme in terms of robustness. In this way, the watermark will be introduced to various well-known attacks, and effectiveness of the scheme is examined. To check the robustness of the image or how much the image is robust against an attack, a normalized correlation is calculated which is a correlation of original and recovered image, given by the following equation:

(9) $$\begin{array}{c} N_{c}\left(W,W^{\prime }\right)=\frac{\sum_{i=0}^{M-1}\sum_{j=0}^{N-1}W\left(i,j\right)W^{\prime }\left(i,j\right)}{\sum_{i=0}^{M-1}\sum_{j=0}^{N-1}{\left[W\left(i,j\right)\right]}^{2}}, \end{array}$$

where *W* and *W*′ represent the original and extracted watermarks of dimension *M* × *N* each, respectively.

#### 4.2.1. Robustness against Salt and Pepper Noise

**Figure 7:**
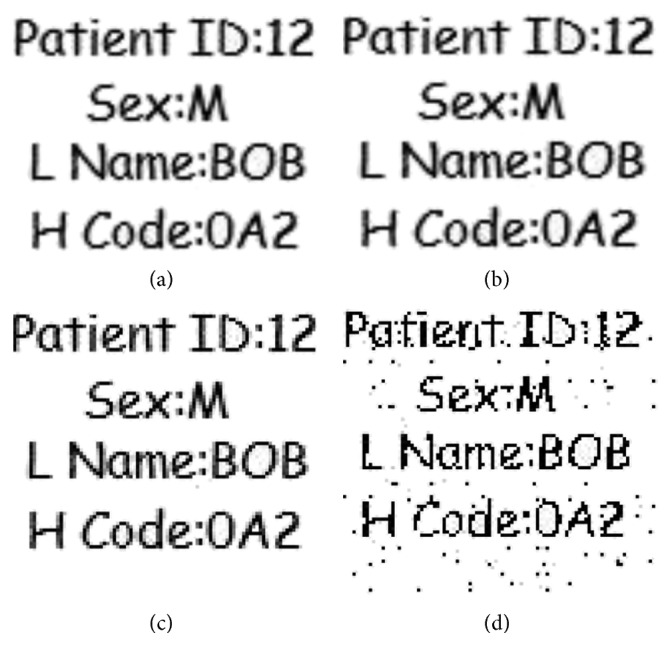
Robustness of the watermark against salt and pepper noise with variance (a) 0.02 (*N*_c_ = 1), (b) 0.03 (*N*_c_ = 1), (c) 0.05 (*N*_c_ = 1), and (d) 0.2 (*N*_c_ = 0.9249).

Robustness of a watermark against salt and pepper noise having variances 0.02, 0.03, 0.05, and 0.2, respectively, are considered in this experiment, and objective measures for these variances are shown in Figure 7 with their extracted watermarks. As we can see that when noise variance was enhanced to 0.2, the watermark is still clearly detectable; this clearly demonstrates the robustness of our proposed scheme. The correlation analysis is given in Table 3. It shows that with variances 0.02, 0.03, and 0.05, there is no effect on image normalized correlation *N*_c_ while in the case of 0.2, there is 0.0571 amount of degradation in the correlation value and still the image is very much recognizable by the naked eye.

#### 4.2.2. Robustness against Speckle Noise

**Figure 8:**
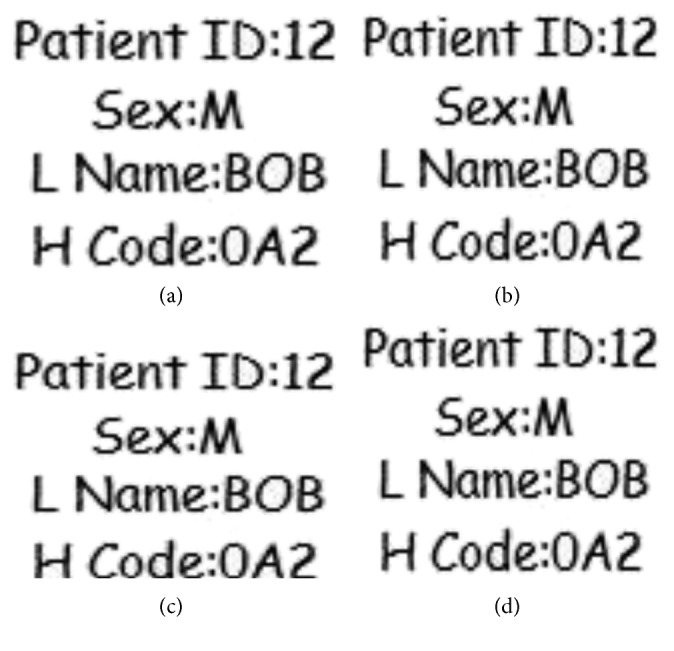
Robustness of the watermark against speckle noise with variances (a) 0.02, (b) 0.03, (c) 0.05, and (d) 0.2.

Robustness of the watermark against speckle noise having variances 0.02, 0.03, 0.05, and 0.2 is considered in this experiment, and objective measures for these variances are shown in Figure 8. The correlation analysis is given in Table 4. It shows that for all the variances of speckle noise taken (0.02, 0.03, 0.05, and 0.2), there is no effect on image normalized correlation; hence there is no significant degradation in the image. So, the scheme is highly robust against the speckle noise.

#### 4.2.3. Robustness against Gaussian Noise

**Figure 9:**
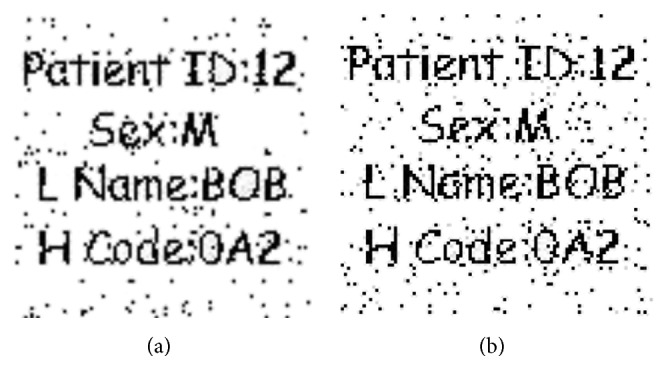
Robustness of the watermark against speckle noise with variances (a) 0.03 and (b) 0.05.

Robustness of the watermark against Gaussian noise having variances 0.03 and 0.05 is considered in this experiment. *N*_c_ and hash for these variances are calculated and are given in Tables 5 and 8, respectively, while the recovered watermark is given in Figure 9.

#### 4.2.4. Robustness against Median Filtering

Robustness of the watermark against median filtering with window sizes of 3 × 3 and 5 × 5 is considered.

**Figure 10:**
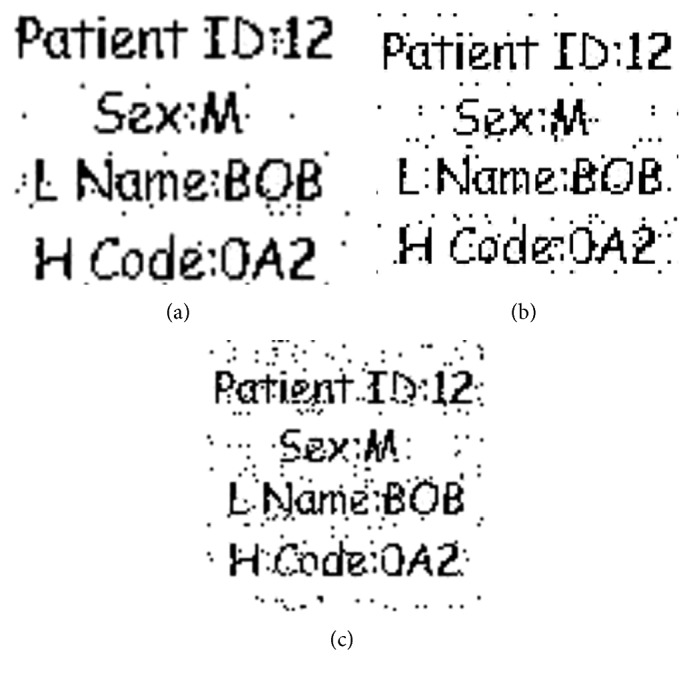
Robustness of the watermark against median filtering with window sizes (a) 3 × 3, (b) 5 × 5, and (c) 7 × 7.

Effects of these filters are shown in Figure 10.

The correlation analysis is given in Table 6. It shows that with the window size of 3 × 3, the effect on image correlation is 0.0739, while in case of window size 5 × 5, there is 0.0179 amount of degradation in the correlation value and hence the image is very much recognizable.

#### 4.2.5. Robustness against Tampering

**Figure 11:**
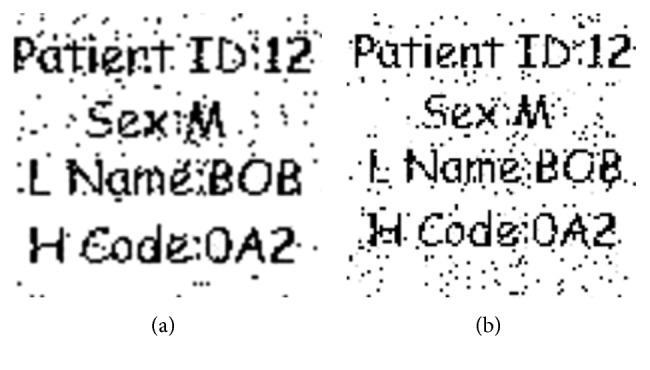
Robustness of the watermark against tampering: (a) 9 bits and (b) 18 bits.

Robustness against tampering is demonstrated, as shown in Figure 11. A total of 9 bits were modified in RONI part of the watermarked image and the watermark is extracted. The watermark is still recognizable as shown in Figure 11(a). When the corrupted bits were of 18 bits, the watermark is still readable as shown in Figure 11(b), which shows the high robustness of the proposed scheme.

The correlation analysis is given in Table 7. It shows that with the tampering of 9 bits, the effect on image correlation is 0.0144, while in case of 18 bits tampering, there is 0.0917 amount of degradation in the correlation value and still the image is very much recognizable.

#### 4.2.6. Robustness against Rotation

**Figure 12:**
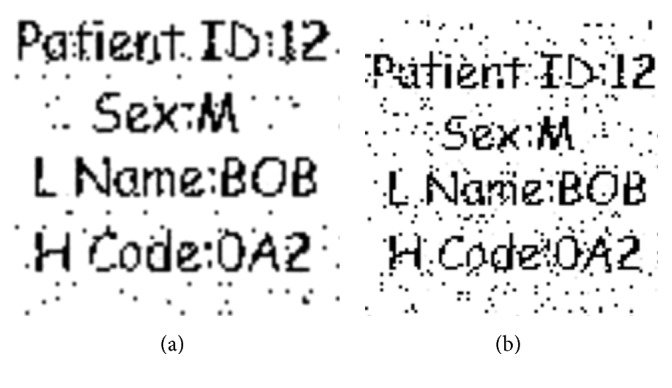
Robustness of the watermark against rotation: (a) 2 degrees and (b) 8 degrees.

Robustness of the watermark against rotation attack of 2 and 8 degrees is shown in Figures 12(a) and 12(b), respectively. The experiment shows that the watermark is still recognizable after the rotation attack of 2 degrees, as shown in Figure 12(a). Similarly, when the watermarked image is again rotated with 8 degrees, the watermark is still detectable as shown in Figure 12(b) which demonstrates the high robustness of the proposed scheme against rotation. The normalized correlation is 0.9856 and 0.9752 which corresponds to a degradation of 0.0144 and 0.0248, respectively, which is given in Table 8.

The values given in Tables 3–8 show that the proposed scheme exhibits a significant level of robustness against the given attacks. This is how we made the watermark, which comprises the patient information, robust. This is accomplished by the product codes used in the proposed scheme, and this scheme exhibits good error correction capability.

### 4.3. Fragility Analysis

The proposed scheme offers high degree of fragility for medical image and high robustness against several attacks to safeguard the watermark. Table 8 shows the fragility analysis of the proposed scheme for all the aforementioned given attacks. In this regard, the only check is comparison of the recovered hash with the original embedded hash of the image. This is given by [a16c4d371f512de40f836428ea2541fe]. From Table 9, it is apparent that the recovered hash after any of the attacks is totally different from the original hash. It shows that the proposed scheme provides a high level of fragility to the medical images.

### 4.4. Imperceptibility Analysis

**Figure 13:**
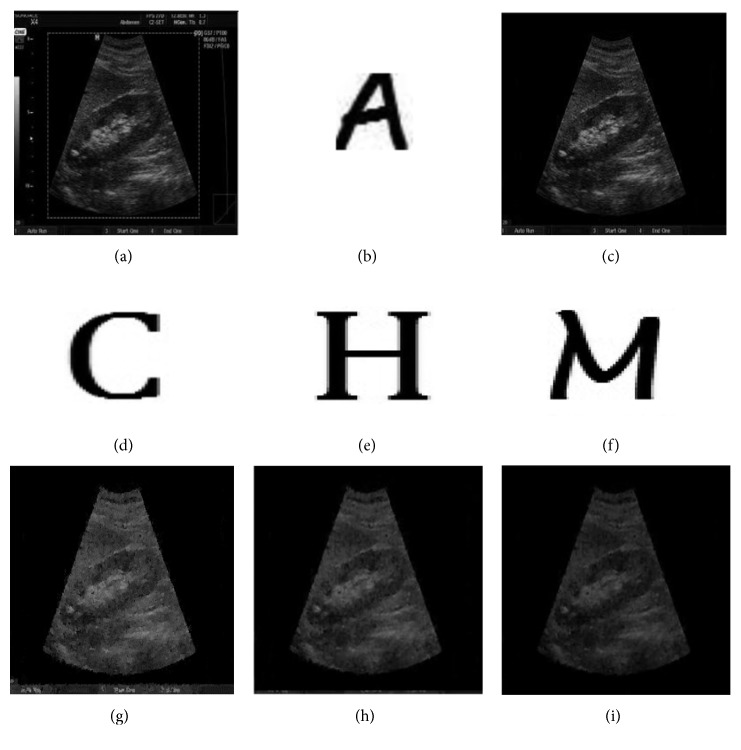
(a) Original host ultrasound image (b, d, e, f), watermark logos (c, g, h, i), and watermarked images. (a) Original ultrasound image. (b) Watermark logo (30 × 30). (c) Watermarked image with logo “A,” PSNR = 42.4 dB. (d) Watermark logo (40 × 40). (e) Watermark logo (50 × 50). (f) Watermark logo (60 × 60). (g) Watermarked image with logo “C,” PSNR = 38.6 dB. (h) Watermarked image with logo “H” PSNR = 32.8 dB. (i) Watermarked image with logo “M,” PSNR = 30.2 dB.

To show the efficiency of the proposed scheme in terms of imperceptibility, a different set of experiments are conducted. Same greyscale ultrasound image of size 194 × 259 pixels is taken as host image and the watermark logos “A,” “C,” “H,” and “M” were considered having sizes 30 × 30, 40 × 40, 50 × 50, and 60 × 60, respectively, for different payload analysis.  Figure 13(a) shows the original ultrasound image with rectangular selected ROI. Figures 13(b) and 13(c) show the watermark logo ‘A,' the watermarked image. Figures 13(d)–13(f) show watermark logos “C,” “H,” and “M,” respectively. Consequently, Figures 13(g)–13(i) contain watermarked images using logos “C,” “H,” and “M”. Hash of the image is given with the figure caption.

As far as the perceptual quality of the watermarked images is concerned, from the naked eye test, the ROI area does not seem to be much affected, however, the RONI is changed. The peak signal to noise ratio (PSNR) values of the watermarked images (which is the measure of imperceptibility) are given with them. The worst PSNR is against the largest logo “M,” which is 30.2 dB, which is acceptable practically [1]. PSNR can be given by the following equation:

(10) $$\begin{array}{c} \text{PSNR}=10\;\log_{10}\frac{255^{2}}{\text{MSE}},\\ \text{where, MSE}=\frac{1}{\text{MN}}\sum_{i=0}^{M-1}\sum_{j=0}^{N-1}{\left[f\left(i,j\right)-f^{\prime }\left(i,j\right)\right]}^{2}, \end{array}$$

where *f* and *f*′ are original and watermarked images, respectively, of dimensions *M* × *N*. Comparison of the proposed scheme with [6] in terms of capacity and imperceptibility is given in Table 10. Capacity is taken as a ratio of watermark size to image size. For the cases where capacity is lower than 10%, the scheme in [6] outperforms the proposed scheme in terms of imperceptibility, for example, a PSNR of 249.6 dB achieved at 7.2% capacity. However, for capacity values higher than 10%, proposed scheme outperforms. For example, the maximum capacity demonstrated in [6] is 14.6% with PSNR achieved as 27.4 dB while in the proposed scheme, 42.4 dB PSNR is achieved with 14% capacity. Moreover, for the capacity 42.9%, the proposed scheme exhibits a PSNR of 30.2 dB while in [6], PSNR value of 31.7 dB is achieved for capacity value 13.6%. Hence, for higher capacities, the proposed scheme performs way better.

**Figure 14:**
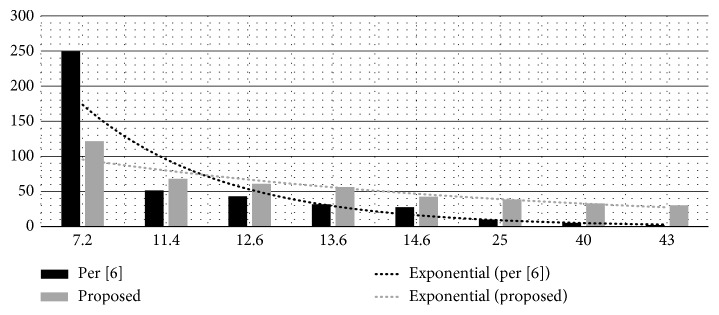
Comparison of the proposed scheme with [6] in terms of capacity and imperceptibility.

Figure 14 shows the comparison of the proposed scheme with the scheme given in [6] in terms of capacity and imperceptibility. The graph is plotted based on Table 10 values. Capacity ratio and PSNR (dB) vales are shown on the *x* and *y* axis, respectively. For capacity values greater than 10%, the proposed scheme outperforms the scheme in [6]. Exponential trend lines show that the proposed scheme exhibits a linear drop while the scheme in [6] exhibits an exponential drop in imperceptibility with increasing capacity ratio. At the capacity ratio above 40%, the proposed scheme exhibits a sustainable imperceptibility while the scheme in [6] shatters.

## 5. Conclusion

A novel digital watermarking scheme for medical images which offers robustness to the watermark information and fragility to the host medical image based on chaotic key, hash function, product codes, and RNS is proposed. In the proposed watermarking scheme, the watermark is robust against a variety of common attacks while the host image is highly fragile against tampering. Added hash function and chaotic key-based embedding ensure the security measures. Embedding locations, duly generated with the help of chaotic system, are highly sensitive to initial conditions. These initial conditions are known at the receiver side that makes possible a blind recovery. The proposed scheme is robust against a variety of attacks because of the error correcting codes utilized. A wide spectrum of simulation results shows the effectiveness of the proposed scheme in terms of robustness, fragility, security, capacity, and imperceptibility. In future, the proposed scheme may be extended for colored (3D) medical images, and to withstand more attacks, host image robustness will make the scheme even interesting.

## Figures and Tables

**Table 1:** Moduli mapping.

Original pair	Substituted pair
(16, 0)	(0, 15)
(16, 1)	(1, 15)
(16, 2)	(2, 15)
(16, 3)	(3, 15)
(16, 4)	(4, 15)
(16, 5)	(5, 15)
(16, 6)	(6, 15)
(16, 7)	(7, 15)
(16, 8)	(8, 15)
(16, 9)	(9, 15)
(16, 10)	(10, 15)
(16, 11)	(11, 15)
(16, 12)	(12, 15)
(16, 13)	(13, 15)
(16, 14)	(14, 15)

**Table 2:** Recovered hash values.

Initial conditions	Recovered hash
*x*(0) = 0.25, *r* = 3.58	a16c4d371f512de40f836428ea2541fe
*x*(0) = 0.25001, *r* = 3.58	f838ea642a16c4d382706e5a9254c83a

**Table 3:** Robustness values against salt and pepper noise.

Variance	*N*_c_ (proposed technique)
0.02	1
0.03	1
0.05	1
0.2	0.9429

**Table 4:** Robustness values against speckle noise.

Variance	*N*_c_ (proposed technique)
0.02	1
0.03	1
0.05	1
0.2	1

**Table 5:** Robustness values against Gaussian noise.

Variance	*N*_c_ (proposed technique)
0.03	0.9261
0.05	0.9204

**Table 6:** Robustness values against median filtering.

Window size	*N*_c_ (proposed technique)
3 × 3	0.9261
5 × 5	0.9204
7 × 7	0.9156

**Table 7:** Robustness values against tampering.

Tampered bits	*N*_c_ (proposed technique)
9	0.9856
18	0.9083

**Table 8:** Robustness values against rotation.

Rotation (in degrees)	*N*_c_ (proposed technique)
2	0.9856
8	0.9752

**Table 9:** Received hash after various attacks.

Attack type	Attack parameter	Recovered hash
Salt and pepper noise	Variance 0.02	a16c4d382706e5a92f838ea64254c83a
	Variance 0.03	82706e5a9254cf838ea642a16c4d383a
	Variance 0.05	f86e5a9254c8338ea642a16c4d38270a
	Variance 0.2	d382706e5a838ea642a16c492f54c83a

Speckle noise	Variance 0.02	834d3827f068ea642a16ce5a9254c83a
	Variance 0.03	f38ea642a54c8316c4d8382706e5a92a
	Variance 0.05	8ea642a16c4d38270f836e5a9254c83a
	Variance 0.2	f83d83382706e5a9254c8ea642a16c4a

Gaussian noise	Variance 0.02	ea5a9254c8642a16c4d382706f838e3a
	Variance 0.05	fa16c4d382706e5838ea642a9254c83a

Median filtering	Window size 3 × 3	8ea642a16c4d382f83706e5a9254c83a
	Window size 5 × 5	f8 ea642a16c4d38382706e5a9254c83a
	Window size 7 × 7	f8c454c83d382706e5a9238ea642a16a

Tampering	9 bits	f838ea69254ca16c4d382706e5a83abb
	18 bits	f42a16c4d3829838ea6706e5a254bab01

Rotation	2 degrees	8a642a16c4d382f83706e5a9e254c83a
	8 degrees	fc4d38382706e5a9254c838ea642a16a

**Table 10:** Imperceptibility versus capacity comparison.

Capacity (%)	PSNR (dB) [6]	Capacity (%)	PSNR (dB) (proposed)
7.2	249.6	7	121.6
11.4	51.5	**11**	67.9
12.6	42.9	**12.4**	60.7
13.6	31.7	**13.2**	56.3
14.6	27.4	**14**	42.4
25	10	**25.5**	38.6
40	5	**39.9**	32.8
43	2	**42.9**	30.2
